# The effects of self-efficacy in managing the disease and disease adaptation levels of Familial Mediterranean Fever (fmf) patients on satisfaction with life: a web-based cross-sectional study

**DOI:** 10.1186/s13023-025-03931-w

**Published:** 2025-08-21

**Authors:** Rahime Nur Demir, Ramazan Kiraç, Fatma Çiftçi Kiraç

**Affiliations:** 1 Bayburt University, Bayburt, Turkey; 2 Kahramanmaraş Sütçü Imam University, Kahramanmaraş, Turkey

**Keywords:** Familial Mediterranean Fever, Self-Efficacy, Satisfaction with life, Chronic disease

## Abstract

**Background:**

Familial Mediterranean Fever (FMF) is an auto inflammatory disease often accompanied by fever and serositis attacks in which peritoneum, pleura, synovium, and rarely pericardium are spared. In the study, the effects of self-efficacy in managing the disease and disease adaptation levels of FMF patients on satisfaction with life were examined.

**Methods:**

This observational cross-sectional study was conducted using a web-based questionnaire sent via Facebook and Instagram FMF groups between February 1, 2024 and April 25, 2024. The population of this study consisted of patients diagnosed with FMF at least 1 year ago in Türkiye.

**Findings:**

The mean self-efficacy score of FMF patients in managing chronic diseases was found to be 4.67. According to the results of the study, physical adaptation was determined to be the highest adaptation in FMF patients. This is followed by psychological adaptation and social adaptation. In general, the scores the patients obtained regarding disease adaptation and its sub-dimensions were found to be close to the average value. The satisfaction with life scores of the patients were found to be below the average.

**Conclusion:**

This study revealed that the self-efficacy and disease adaptation levels of FMF patients in Türkiye affect their satisfaction with life.

## Introduction

Familial Mediterranean Fever (FMF) is an auto inflammatory disease often accompanied by fever and serositis attacks in which peritoneum, pleura, synovium, and rarely pericardium are spared [34]. The disease was first diagnosed in 1908 in a 16-year-old girl of Jewish origin who complained of recurrent fever and abdominal pain and was named unusual recurrent peritonitis [18]. The definition of “Familial Mediterranean Fever” was first used by an Israeli researcher Heller in 1958 [15]. FMF, an autosomal recessive disease, affects more than 100,000 people worldwide and is therefore the most common among hereditary periodic fevers [22]. FMF symptoms occur in the first decade of life in approximately 60% of patients and before the age of 20 in 90% of patients [30]. Abdominal attacks are the most common symptoms in approximately 95% of patients [26]. The duration of attacks generally varies between 2 and 4 days, but they can also last longer or shorter. Although the factors triggering attacks are generally unknown, infections and stress are thought to play an important role [35]. The disease occurs frequently especially in the people living in the Eastern Mediterranean basin and in Sephardic Jews, Armenians, Arabs, and Turks [19]. The incidence of FMF in Türkiye is known to be 1/1000, and the carrier rate of the disease was reported as 15–34% in various studies [10]. A study conducted in the northwest of Türkiye revealed a prevalence of 6:10,000 [6]. These striking differences show that FMF is not evenly distributed even in geographies at-risk such as Türkiye and point to the genetic heterogeneity of these populations [25]. FMF is a common health problem in Türkiye; therefore, early diagnosis of it is important for the treatment of the disease and prevention of possible complications [35]. Facilitating the health care of patients with FMF, managing the disease, and determining the disease adaptation levels of individuals can be effective in increasing both the effectiveness of health interventions for patients and their quality of life and satisfaction with life.

## Materials and methods

### Research model and hypothesis

This descriptive study aims to reveal the effects of self-efficacy in managing the disease and disease adaptation levels of FMF patients on satisfaction with life. The study tries to find answers to various questions as below;

1. Does self-efficacy in managing the disease of FMF patients affect their satisfaction with life?
2. Does disease adaptation of FMF patients affect their satisfaction with life?

**Fig. 1:**
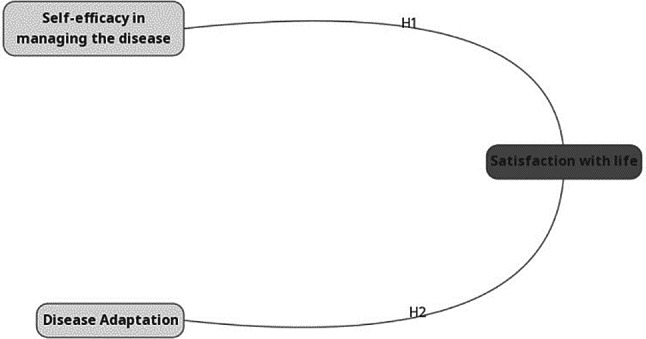
Research model

The research hypotheses developed in line with the research purposes are listed below and the research model is shown below Fig. 1.

H1: FMF patients’ self-efficacy in managing the disease affects their satisfaction with life.

H2: FMF patients’ disease adaptation affects their satisfaction with life.

### Study design and participants

This observational cross-sectional study was conducted using a web-based questionnaire sent via Facebook and Instagram FMF groups between February 1, 2024 and April 25, 2024. The population of this study consisted of patients diagnosed with FMF at least 1 year ago in Türkiye. The sample of the study consisted of all FMF patients receiving outpatient or inpatient treatment. In our study, participants were reached through BEFEMDER (Behçet and Familial Mediterranean Fever Patients’ Association). The necessary permissions were obtained from the mentioned association. The FMF diagnosis was not directly verified by the researchers; however, the association exclusively includes diagnosed individuals and requires the submission of a medication usage report for membership. Additionally, it was assumed that patients had previously been diagnosed by relevant physicians.

Convenience sampling method was used in the participant selection of this study. This method involves the researcher selecting potential participants based on ease of access [21]. The minimum required sample size of this study was determined as 385 with a 5% margin of error, 95% confidence interval, 80% power, and 0.05 significance level in the power analysis, and 423 people participated in the study. The inclusion criteria of the study included being diagnosed with FMF at least 1 year ago, being 18 years of age or older, being physically and cognitively able to answer the questionnaire, using web applications (Facebook, Instagram), and agreeing to participate in the study. All participants who met the inclusion criteria were invited to the study and the study was conducted with volunteers.

## Data collection tools

### Detailed information about the scales used in the study is provided below

#### The Self-Efficacy to manage chronic diseases

The scale developed by [23] is made up of 6-items. The scale is rated as 10 points ranging from “not at all confident” to “totally confident”. The score of the scale is the mean of 6 items, and a high mean score indicates high self-efficacy. The Turkish validity and reliability study of the scale was conducted by İncirkuş and Nahcivan in 2020 [17].

#### Assessing adaptation to chronic illnesses

The scale, developed by Atık and Karatepe in 2018, consists of three sub-dimensions as physical (1,9,10,13,14,15,16,18,22,23,24), social (2,3,5,7,17,19,25), and psychological (4,6,8,11,12,20,21) adaptation and 25 items. The scale is a 5-point Likert type ranging from “strongly disagree” to “strongly agree”. While the items 1, 2, 3, 4, 7, 8, 9, 10, 11, 13, 14, 15, 16, 18, 21, 22, 23 are scored normal (as 1,2,3,4,5), the items 5, 6, 12, 17, 19, 20, 24, 25 are reverse (as 5,4,3,2,1) scored. The total score obtained from the scale is 125. An increase in the scores obtained from the sub-dimensions and/or the entire scale means that disease adaptation levels of the patients also increase [2].

#### Satisfaction with life

The scale, developed by Diener in 1984, is a 7-point Likert type scale consisting of a single dimension and five items. The scale items are scored between “strongly disagree” and “strongly agree”. The Turkish validity and reliability study of the scale was conducted by Dağlı and Baysal in 2016. As a result of the Turkish validity and reliability study, the seven-point Likert was reduced to five-point Likert [7, 9].

### Ethical considerations

In order to conduct the study, the necessary permission was received from Bayburt University Non-invasive Research Ethics Committee, dated 23.02.2024 and with decision number 2023/03–47. An informed consent option was provided on the first page of the online questionnaire. On the first page of the questionnaire, it was explained to all participants electronically that they were willing to participate in the study and that they could leave the study at any time. The data were collected in a way that did not reveal the identities of the patients. No financial support was received from any institution for this study, and no financial payment was made to the patients who participated in the study. The study was conducted in accordance with the principles of the Declaration of Helsinki.

### Data analysis

All data obtained within the scope of the study were analyzed using the SPSS (Statistical Package for the Social Sciences) 22.0 package program [16]. Within the scope of the study, firstly, validity and reliability studies of The Self-Efficacy to Manage Chronic Diseases, Assessing Adaptation to Chronic Illnesses, and Satisfaction with Life were conducted. It was determined that the fit indices for The Self-Efficacy to Manage Chronic Diseases were at a good fit level (CMIN/DF:2.956; RMESA:0.068; GFI:0.982; NFI:0.977; CFI:0.984). It was determined that the fit indices for Assessing Adaptation to Chronic Illnesses were at an acceptable level (CMIN/DF: 3.123.; RMESA:0.071; GFI:0.841; NFI:0.768; CFI:0.827). It was determined that the fit indices for Satisfaction with Life were at a good fit level (CMIN/DF:1.031; RMESA:0.009; GFI:0.996; NFI:0.978; CFI:0.999). Additionally, the Cronbach Alpha value was detected as 829 for The Self-Efficacy to Manage Chronic Diseases; 0.809 for Assessing Adaptation to Chronic Illnesses; and 0.879 for Satisfaction with Life.

Within the scope of the study, descriptive statistics were obtained and multivariate regression analyzes were performed. Durbin Watson Coefficient and Variance Inflation Factor (VIF) were calculated to determine whether multicollinearity and autocorrelation existed in the linear regression models. In all statistical tests, the alpha level was taken as 0.05.

## Findings

### Descriptive findings

**Table 1:** Patient characteristics

Patient Characteristics		*N*	(%)
Gender	Male	111	26.2
	Female	312	73.8
Age	18–24	42	9.9
	25–31	83	19.6
	32–38	116	27.4
	39–45	113	26.7
	46 and above	69	16.3
Marital Status	Married	311	73.5
	Single	112	26.5
Disease Duration	0–5 years	65	15.4
	6–10 years	57	13.5
	11–15 years	75	17.7
	16–20 years	64	15.1
	21 years and above	162	38.3
Regular Check-Up Status	I Go for Check-ups Regularly	277	65.5
	I Do Not Go to Check-ups Regularly	146	34.5
Health Assessment Status	Good	110	26
	Moderate	266	62.9
	Bad	47	11.1

Table 1 shows the results of the socio-demographic variables of patients. More than half of the patients are women (73.8%), 27.4% are between the ages of 32–38, 26.7% are between the ages of 39–45, 19.6% are between the ages of 25–31, 16.3% are 46 years old and over, and 9.9% are between the ages 18–24. While more than half of the patients (73.5%) are married, 38.3% have a disease duration of 21 years or more. While 65.5% of the participants go for regular check-ups, 62.9% evaluate their health as moderate.

**Table 2:** Mean, standard deviation, and correlation coefficients of variables used in the study

	Mean	sd.	1	2	3	4	5	6
(1)Self-efficacy in Managing the Disease	4.67	1.89	1	0.532**	0.350**	0.469**	0.492**	0.417**
(2)Disease Adaptation	3.10	0.51	0.532**	1	0.785**	0.818**	0.818**	0.564**
(3)Physical Adaptation	3.36	0.57	0.350**	0.785**	1	0.370**	0.412**	0.351**
(4)Social Adaptation	2.87	0.74	0.469**	0.818**	0.370**	1	0.669**	0.502**
(5)Psychological Adaptation	2.92	0.62	0.492**	0.818**	0.412**	0.669**	1	0.545**
(6)Satisfaction with Life	2.68	0.90	0.417**	0.564**	0.351**	0.502**	0.545**	1

**p < .001; *p < .05

The mean self-efficacy score of FMF patients in managing chronic diseases is 4.67. In general, it can be said that the mean self-efficacy score of the patients in managing the disease is high. The mean score for assessing adaptation to chronic diseases is 3.10. When the sub-dimensions of disease adaptation are examined, physical adaptation has the highest mean score with 3.36, followed by psychological adaptation with 2.92 and social adaptation with 2.87. It can be said in general that the scores they received regarding disease adaptation and its sub-dimensions are close to the average value. The mean satisfaction with life score is 2.68. In general, the mean satisfaction with life score of the patients is below the average (Table 2).

A positive and moderate relationship (*r* = .532; *p* < .001) was detected between self-efficacy in managing the disease and disease adaptation and a positive and moderate relationship (*r* = .417; *p* < .001) was detected between self-efficacy in managing the disease and satisfaction with life. Moreover, a positive and moderate relationship (*r* = 564; *p* < .001) was detected between disease adaptation and satisfaction with life.

### Regression analysis

**Table 3:** Regression analysis results to predict the effects of Self-Efficacy in managing the disease and patient evaluations for disease adaptation on satisfaction with life

Predicted Variables	Predicted Variables	β	t	*p*	VIF
*Satisfaction with Life*	*Self-Efficacy in Managing the Disease*	0.417	9.421	< 0.001	1.000
*R* = .417;R^2^ = 0.174; F = 88.750;*p* = .001; Durbin Watson = 1.848					
*Satisfaction with Life*	*Disease Adaptation*	0.564	14.010	< 0.001	1.000
*R* = .564;R^2^ = 0.318; F = 196.286;*p* = .001; Durbin Watson = 1.943					

Table 3 gives the regression analysis results showing the effects of self-efficacy in managing the disease and disease adaptation of patients on satisfaction with life. The fact that the Durbin Watson coefficients of the created regression model are below 2.5 and the Variance Inflation Factor (VIF) is less than 10 indicate that there is no multicollinearity and autocorrelation. The results reveal that self-efficacy in managing the disease has a statistically significant explanatory effect on satisfaction with life (β = 0.417; F = 88.750; *p* < .001). Self-efficacy in managing the disease explains 17.4% of the total variance in satisfaction with life of patients. Based on this result, *“*Hypothesis 1*”*, which states that *“FMF patients’ self-efficacy in managing the disease affects their satisfaction with life”*, was confirmed.

As the results in Table 3 show, disease adaptation was found to have a significant effect on satisfaction with life (β = 0.564; F = 196.286; *p* < .001). Disease adaptation explains 31.8% of the total variance in satisfaction with life of patients. Based on this result, *“*Hypothesis 2*”*, which states that *“FMF patients’ disease adaptation affects their satisfaction with life”*, was confirmed.

## Discussion

In the study, the effects of self-efficacy in managing the disease and disease adaptation levels of FMF patients in Türkiye on satisfaction with life were examined. According to the study results, disease adaptation and self-efficacy in managing the disease of individuals affect satisfaction with life. Consistent with previous studies on FMF patients, more than half of the participants included in the study were women (73.8%) and 27.4% of the patients were between the ages of 32–38 [4, 8, 13, 36, 38, 40]. The mean self-efficacy score of FMF patients in managing chronic diseases was found to be 4.67. In general, it can be said that the mean self-efficacy score of the patients in managing the disease is high. Self-efficacy is a critical concept in chronic disease management, and is important in the evaluation and management of patients with chronic diseases. Measuring self-efficacy in patients is useful in planning patient education programs because identifying areas where the patient has low self-efficacy helps target self-management education to the patient [11]. Moreover, measuring self-efficacy is an important indicator in predicting important health outcomes such as hospitalizations or health-related quality of life. FMF patients are advised to make lifestyle changes to manage their health condition and reduce symptom distress. Success in changing lifestyle depends partly on a person’s self-efficacy belief [5]. Therefore, self-efficacy is an important factor in coping with the challenges and demands caused by FMF disease. Improving self-management and self-efficacy improves quality of life, coping skills, symptom management, and disability. Moreover, it can play an important role in reducing health expenditures and service use [12]. According to the results of the study, physical adaptation was determined to be the highest adaptation in FMF patients. This is followed by psychological adaptation and social adaptation. In general, the scores the patients obtained regarding disease adaptation and its sub-dimensions were found to be close to the average value. A moderate level of disease adaptation can be said to reduce the discomfort of FMF patients during the disease and treatment, increase their compliance with the treatment, and reduce the negative emotions and reactions caused by the disease process [29]. The higher the level of adaptation and acceptance of the disease in patients, the more self-sufficient the patient is, the more active they are, and the more they generally accept the necessity of treatment [32]. In a study on FMF patients’ acceptance of the disease, a moderate acceptance mean score was found, similar to this study [29]. In another study, the compliance rate for regular follow-up of FMF patients was found to be 48.5% [28]. FMF is a hereditary disorder that requires close monitoring and affects individuals throughout life. Non-compliance with FMF treatment and therefore inadequate disease management can lead to life-threatening complications such as secondary amyloidosis even in asymptomatic patients [28].

The satisfaction with life scores of the patients were found to be below the average. The findings obtained from studies in the literature on satisfaction with life and quality of life of FMF patients are consistent with those of this study [1, 8, 13, 24, 27, 33, 37, 38]. Satisfaction with life and quality of life are important in chronic diseases such as FMF and reflect successful disease management. Knowing the deficiencies in satisfaction with life and quality of life in FMF disease is important for disease management due to the course of these chronic disease [37]. FMF symptoms appear in approximately 60% of patients in the first decade of life, and the onset of the disease at an early age makes it difficult to closely follow-up patients [30]. The treatment goals in FMF are to improve the individual’s satisfaction with life and quality of life, reduce the frequency, severity, and duration of the disease, and prevent long-term damage by minimizing chronic/subclinical inflammation [38].

Self-efficacy in managing the disease and disease adaptation of FMF patients affect their satisfaction with life. Physical and mental problems and disabilities experienced by individuals as a result of chronic diseases reduce the independence of individuals and force individuals and their families to make various lifestyle changes because of the disease [3]. Self-efficacy, which is an individual variable, is an important motivational resource in the management of chronic disorders such as FMF, initiating and maintaining health behaviors, and in case of lifestyle changes [20]. As individuals’ self-efficacy increases, their adaptation to chronic diseases becomes easier. In a study conducted with individuals with more than one chronic disease, it was found that as individuals’ adaptation to chronic diseases increases, their self-efficacy increases [14]. Satisfaction with life and quality of life of patients with a high level of disease adaptation is also positively affected. FMF is a chronic auto inflammatory disease that negatively affects satisfaction with life of patients. The course of this disease and the burden of lifelong medications have an impact on the quality of life and satisfaction with life outcomes in FMF patients [33]. Regarding this, if self-efficacy levels of patients are determined and deficient areas are supported, adaptation process of patients to the disease will be accelerated and their satisfaction with life will be increased. Lack of adherence to the disease and medication compliance leads to more frequent attacks and more frequent visits to doctors and hospitals in the long term [31]. Moreover, the severity and frequency of attacks cause absenteeism from work/school, loss of labor force and increased treatment costs per patient [38]. In order to facilitate and promote patients’ compliance with medication and disease, it is recommended to strengthen patient-physician communication, conduct interventions aimed at improving medication compliance and use a person-centered approach to promote self-efficacy [39]. Healthcare professionals working with patients with FMF are recommended to provide regular individual or group-based individualized education to these patients. Adherence to FMF and reduction of disease severity can only be achieved through a planned education program. It is important to develop education and rehabilitation programs and provide psychosocial support in line with the needs of patients.

Limitations of the study include sampling bias, inherent limitations of the cross-sectional design and the use of self-reported data. Other limitations, include the inability to investigate other potential influencing factors such as the severity of FMF, medication adherence and comorbidities.

## Conclusion

This study revealed that the self-efficacy and disease adaptation levels of FMF patients in Türkiye affect their satisfaction with life. The findings reveal the importance of increasing satisfaction with life levels of FMF patients. Additionally, this study emphasizes the importance of developing education and rehabilitation programs and the need for psychosocial support to improve satisfaction with life.

## Data Availability

To protect study participant privacy, data cannot be shared openly.

## Abbreviations

FMF: Familial Mediterranean Fever

## References

[1] Alayli G, Durmus D, Ozkaya O, Sen HE, Nalcacioglu H, Bilgici A, Kuru O. Functional capacity, strength, and quality of life in children and youth with Familial mediterranean fever. Pediatr Phys Ther. 2014;26(3):347–52. 24979093 10.1097/PEP.0000000000000052

[2] Atik D, Karatepe H. Scale development study: adaptation to chronic illness. Acta Med Mediterranea. 2016;32(1):135–42. 10.19193/0393-6384_2016_1_21.

[3] Bilgiç Ş, Pehlivan E. Kronik Hastalığa Sahip Bireylerin Hastalığa Uyumunun Yaşam kalitesi Ile Ilişkisi. Samsun Sağlık Bilimleri Dergisi. 2023;8(1):63–76.

[4] Bodur H, Yurdakul FG, Duruöz MT, Cay HF, Ülkü UÇAR, Keskin Y, Sezer I. Familial mediterranean fever: Health-related quality of life and associated variables in a National cohort. Archives Rheumatol. 2021;36(2):159. 10.46497/ArchRheumatol.2021.8215 PMC8418764 34527919

[5] Bonsaksen T, Lerdal A, Fagermoen MS. Factors associated with self-efficacy in persons with chronic illness. Scand J Psychol. 2012;53(4):333–9. 10.1111/j.1467-9450.2012.00959.x. 22680700 10.1111/j.1467-9450.2012.00959.x

[6] Çakır N, Pamuk ÖN, Derviş E, İmeryüz N, Uslu H, Benian Ö. … enocak, M. (2012). The prevalences of some rheumatic diseases in western Turkey: Havsa study. *Rheumatology international*, *32*, 895–908. 10.1007/s00296-010-1699-4 21229358

[7] Dağlı A, Baysal N. Yaşam Doyum ölçeği̇ni̇n türkçe’ye uyarlanmasi:geçerli̇k ve güveni̇rli̇k Çalişmasi. Elektronik Sosyal Bilimler Dergisi. 2016;15(59):1250–62. 10.17755/esosder.263229.

[8] Deger SM, Ozturk MA, Demirag MD, Aslan S, Goker B, Haznedaroglu S, Onat AM. Health-related quality of life and its associations with mood condition in Familial mediterranean fever patients. Rheumatol Int. 2011;31:623–8. 20049448 10.1007/s00296-009-1334-4

[9] Diener E. (1984). Subjective well-being. In Psychological Bulletin (Vol. 95, Issue 3, pp. 542–575). American Psychological Association. 10.1037/0033-2909.95.3.542 6399758

[10] Erden G, Bal C, Güngör Torun O, Uğuz N, Yıldırımkaya MM. (2008). Ailesel Akdeniz Ateşi (Fmf) Düşünülen Olgularda MEFV gen Mutasyonları Sıklığının incelenmesi.

[11] Frei A, Svarin A, Steurer-Stey C, Puhan MA. Self-efficacy instruments for patients with chronic diseases suffer from methodological limitations-a systematic review. Health Qual Life Outcomes. 2009;7:1–10. 19781095 10.1186/1477-7525-7-86 PMC2761851

[12] Grady PA, Gough LL. Self-management: a comprehensive approach to management of chronic conditions. Am J Public Health. 2014;104(8):e25–31. 24922170 10.2105/AJPH.2014.302041 PMC4103232

[13] Guler T, Garip Y, Dortbas F, Dogan YP. Quality of life in Turkish patients with Familial mediterranean fever: association with fatigue, psychological status, disease severity and other clinical parameters. Egypt Rheumatologist. 2018;40(2):117–21.

[14] Güney A, Basit G. Relationship between disease adaptation and Self-Efficacy for disease management in individuals with multiple chronic diseases. Gevher Nesibe J Med Health Sci. 2023;8(4):1191–201. 10.5281/zenodo.10049232.

[15] Heller H, Sohar E, Sherf L. Familial mediterranean fever. AMA Archives Intern Med. 1958;102(1):50–71. 10.1001/archinte.1958.00260190052007 13558745

[16] Corp IBM, N. IBM SPSS statistics for windows. Version 25.0. NY: IBM corp Armonk; 2017.

[17] İncirkuş K, Özkan Nahcivan N. Validity and reliability study of the Turkish version of the self-efficacy for managing chronic disease 6-item scale. Turk J Med Sci. 2020;50(5):1254–61. 10.3906/sag-1910-13. 32336077 10.3906/sag-1910-13 PMC7491285

[18] Janeway TC, Mosenthal HO. AN UNUSUAL PAROXYSMAL SYNDROME, PROBABLY ALLIED TO RECURRENT VOMITING: WITH A STUDY OF THE NITROGEN METABOLISM. Arch Intern Med. 1908;2(3):214–25.

[19] Kasapçopur Ö, Arısoy N. Ailesel Akdeniz Ateşi ve diğer Otoenflamatuar Hastalıklar Derleme. Türk Pediatri Arşivi. 2006;41(1):9–17.

[20] Kaynak GK. Kronik Hastalıklarda Öz yeterlilik algısı: türkiye’de gerçekleştirilen Çalışmalar Üzerine Bir Derleme. Oltu Beşeri Ve Sosyal Bilimler Fakültesi Dergisi. 2022;3(2):79–86.

[21] Kılıç S. (2013). *Örnekleme Yöntemleri*. *1*, 5455. 10.5455/jmood.20130325011730

[22] Lidar M, Livneh A. Familial mediterranean fever: clinical, molecular and management advancements. Neth J Med. 2007;65(9):318–24. 17954950

[23] Lorig KR, Sobel DS, Ritter PL, Laurent D, Hobbs M. Effect of a self-management program on patients with chronic disease. Effective Clin Practice: ECP. 2001;4(6):256–62. 11769298

[24] Makay B, Unsal E, Arslan N, Varni JW. Health-related quality of life of school-age children with Familial mediterranean fever. Clin Exp Rheumatol. 2009;27(2 Suppl 53):S96–101. 19796543

[25] Ozdogan H, Ugurlu S. Familial mediterranean fever. La Presse Médicale. 2019;48(1):e61–76. 30686512 10.1016/j.lpm.2018.08.014

[26] Pasa SR, Altintas A, Devecioglu BL, Cil TMN, Danis R, Isi HL, Ayyildiz O. Familial mediterranean fever gene mutations in the southeastern region of Turkey and their phenotypical features. Amyloid. 2008;15(1):49–53. 18266121 10.1080/13506120701815456

[27] Press J, Neumann L, Abu-Shakra M, Bolotin A, Buskila D. Living with a child with Familial mediterranean fever: does it affect the quality of life of the parents? Clin Exp Rheumatol. 2000;18(1):103–6. 10728454

[28] Bilici Salman R, Babaoglu H, Satiş H, Yapar D, Avanoglu Güler A, Karadeniz H, Ataş N, Haznedaroğlu Ş, Öztürk MA, Göker B, Tufan A. Compliance of Familial mediterranean fever patients with regular Follow-up visits and associated factors. J Clin Rheumatology: Practical Rep Rheumatic Musculoskelet Dis. 2022;28(1):e77–80. 10.1097/RHU.0000000000001632. 10.1097/RHU.0000000000001632 33298810

[29] Şentürk S, Keskin AY, Turan Ş. Investigation of acceptance of illness and religious coping in patients with Familial mediterranean fever in Turkey. J Relig Health. 2022;61(5):3922–39. 35604514 10.1007/s10943-022-01577-7

[30] Sohar E, Gafni J, Pras M, et al. Familial mediterranean fever: A survey of 470 cases and review of the literature. Am J Med. 1967;43:227–53. 10.1016/0002-9343(67)90167-2 5340644

[31] Sönmez HE, Aktay Ayaz N. Ailevi Akdeniz Ateşi Tanısı Ile Takipli Hastalarda Ilaç Uyumunun Hastalık Seyrine Etkisi. Acta Med Nicomedia. 2022;5(1):8–11. 10.53446/actamednicomedia.1054120.

[32] Szafraniec R, Szczuka E, Pawłowska A. Akceptacja choroby Przez Pacjentów z reumatoidalnym Zapaleniem Stawów. Fizjoter Pol. 2012;12(1):39–48.

[33] Thomas MM, El-Bassyouni HT, El-Massieh A, Phoebe M, Hamed K. Health-related quality of life in Egyptian patients with Familial mediterranean fever. Middle East J Med Genet. 2022;11(1):38–45.

[34] Ugan Y, Ermiş F. Ailesel Akdeniz Ateşi. Med J Süleyman Demirel Univ. 2011;18(4):139–43.

[35] Üstebay S, Üstebay DÜ, Yılmaz Y. Familial mediterranean fever. JAREM J Acad Res Med. 2015;5(3):89.

[36] Uzun N, Tarakcı E, Uğurlu S. Physical activity level, sleep, fatigue and quality of life in behçet’s disease and Familial mediterranean fever disease during the Covid 19 pandemic. J Exerc Therapy Rehabilitation. 2023;10(1):37–47.

[37] Yildirim DG, Bakkaloglu SA, Acar ASS, Celik B, Buyan N. Evaluation of quality of life and its associations with clinical parameters in pediatric patients with Familial mediterranean fever. North Clin Istanbul. 2021;8(3):255–60. 10.14744/nci.2020.90093. 10.14744/nci.2020.90093 PMC8240233 34222806

[38] Yildirim D, Vasi I, Tahta E, Kardaş RC, Özkızıltaş B, Küçük H, Tufan A. Factors affecting patient-acceptable symptom States and treatment decision in Familial mediterranean fever. Turk J Med Sci. 2022;52(6):1991–6. 36945975 10.55730/1300-0144.5547 PMC10390155

[39] Yoon S, Kwan YH, Yap WL, Lim ZY, Phang JK, Loo YX, Aw J, Low LL. Factors influencing medication adherence in multi-ethnic Asian patients with chronic diseases in singapore: A qualitative study. Front Pharmacol. 2023;14:1124297. 10.3389/fphar.2023.1124297. 36969865 10.3389/fphar.2023.1124297 PMC10034334

[40] Yüksel F. (2010). *Ailevi akdeniz ateşi hastalarında yaşam beklentisi ve belirleyicileri* (Doctoral dissertation, Dokuz Eylul Universitesi (Turkey)).

